# Response to: Comment on: “Sex-Specific Differences in Running Injuries: A Systematic Review with Meta-Analysis and Meta-Regression”

**DOI:** 10.1007/s40279-021-01549-z

**Published:** 2021-09-04

**Authors:** Karsten Hollander, Jan Wilke, Astrid Zech

**Affiliations:** 1 Institute of Interdisciplinary Exercise Science and Sports Medicine, MSH Medical School Hamburg, Am Kaiserkai 1, 20457 Hamburg, Germany; 2 Institute of Occupational, Social and Environmental Medicine, Goethe University Frankfurt, Frankfurt, Germany; 3 Department of Human Movement Science and Exercise Physiology, Institute of Sport Science, Friedrich Schiller University Jena, Jena, Germany

Dear Editor,

We really appreciate the Letter to the Editor by Nnamani Silva et al. [1], which added valuable information and discussion to our systematic review titled “Sex-specific differences in running injuries: a systematic review with meta-analysis and meta-regression” [2].

The unequal sample size of sexes, with more male runners in road racing events and more female novice runners, emphasizes the need to take a closer look at moderating factors. Generally, detailed reporting of potential effect modifiers is highly encouraged in primary studies to increase the often limited power of meta-regressions. Regardless, in our meta-analysis, the exclusive inclusion of studies with both sexes for the same running background (level) and use of incidences for risk ratio calculation of each study should have reduced the influence of unequal sample size distribution. However, we agree that the combination of studies with different running levels in the same pooled risk ratio calculation may have led to a greater weighting of one running level (towards the level with the higher number of studies). In our meta-regression, we quantified the running level with the competition distance, training duration, and training mileage but cannot completely rule out that a differentiation for the competition level (road racing vs. novice) would have led to different results.

In conclusion, the points raised by Nnamani Silva et al. [1] highlighted another important aspect in the relevant consideration of sex as a variable for equal sampling in addition to the possible impact of sex specificity in the etiology and probably prevention and rehabilitation of running-related injuries.
